# Complete genome sequence of vB_PaBD_211, a lytic *Pseudomonas aeruginosa* phage isolated from poultry sewage in Bangladesh

**DOI:** 10.1128/mra.00893-25

**Published:** 2025-11-24

**Authors:** Morioum Sarkar, Tasnimul Arabi Anik, Sangita Ahmed

**Affiliations:** 1 Department of Microbiology, University of Dhaka 95324, Dhaka, Bangladesh; Portland State University, Portland, Oregon, USA

**Keywords:** bacteriophages

## Abstract

A lytic bacteriophage, vB_PaBD_211, targeting *Pseudomonas aeruginosa* was isolated from poultry sewage in Dhaka, Bangladesh. The 43,381 bp double-stranded DNA genome was sequenced using Illumina MiSeq and assembled *de novo*. The genomic features, absence of virulence or antibiotic resistance genes, and lytic nature suggest its suitability for phage-based therapeutic applications.

## ANNOUNCEMENT

**Fig 1:**
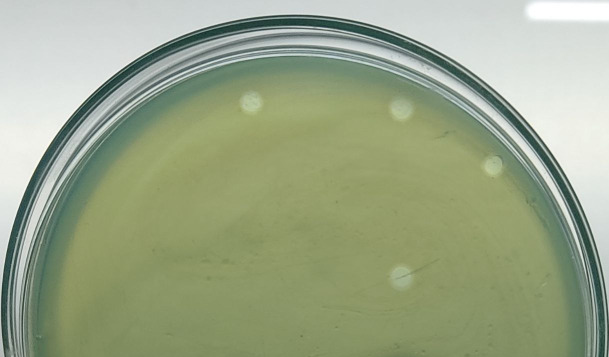
Plaques formed by phage vB_PaBD_211 on *P. aeruginosa* strain DUFMBL1 lawns using the double-layer agar method.

*Pseudomonas aeruginosa* is an opportunistic pathogen associated with severe hospital- and community-acquired infections and increasing multidrug resistance (1, 2). The emergence of lytic bacteriophages as potential therapeutic agents has renewed interest in phage genome characterization (3). To explore phage-based strategies, we isolated a lytic *Pseudomonas aeruginosa* phage, vB_PaBD_211, from poultry sewage collected in the Anandabazar market area, Dhaka (23°43′26″ N, 90°24′14″ E) on 12 October 2024. Samples were centrifuged and sequentially filtered through a 0.22 µm membrane filter. The phage was isolated by adding 100 µL of the filtered sewage to 100 µL of *P. aeruginosa* strain DUFMBL1 in the logarithmic growth phase, followed by single-plaque purification using the double-layer agar assay (Fig. 1) (3). A purified plaque (3–4 mm) was amplified through three successive passages. Lysates were prepared on LB agar by overlaying confluent lawns and flooding with SM buffer after plaques formed (12–18 h); collected buffer was centrifuged (10,000 × *g*, 10 min), filtered (0.22 µm), and used to prepare high-titer stocks (~10⁸ to 10¹¹ PFU/mL) for DNA extraction. Lysates were treated with DNase I/RNase I (80 µg/mL) at 37°C for 3 h prior to PEG precipitation and DNA extraction using a modified protocol of the DNeasy Blood and Tissue Kit (Qiagen) (4). Library preparation was performed using the Illumina DNA Prep kit (Illumina, San Diego, CA, USA), and sequencing was carried out on the Illumina MiSeq platform. A total of 89,528 raw reads (paired) were generated, corresponding to 25.8 Mb of sequencing data (read length 250 bp and coverage 570.23×).

In the sequence analysis, default parameters were used. Raw reads were quality-checked using FastQC (v.0.12) (5) and trimmed with Trimmomatic (v.0.39) (ILLUMINACLIP:TruSeq3-PE.fa:2:30:10, SLIDINGWINDOW:4:20, MINLEN:50) (6). *De novo* assembly was carried out using SPAdes (v.4.2) (7), with the “--careful” option enabled and default k-mer sizes, and assembly quality was evaluated using QUAST (v.5.3.0) (8). Genome annotation was performed with Pharokka (v.1.7.5) (9). Genome completeness and quality were assessed using CheckV (v.1.0.1) (10). NCBI BLASTn was used to determine the taxonomic relationships of the phage (11). The virulence factors and antibiotic resistance factors were detected using the VFDB (v.2.0) (12) and ResFinder (v.4.00) (13) databases, respectively.

The genome of phage vB_PaBD_211 is a linear double-stranded DNA with a length of 43,381 bp, a GC content of 62%, 100% completeness, and 0.00% contamination (Table 1). Linearity was inferred from the assembly structure visualized in Proksee (14). CheckV predicted 53 viral genes and confirmed the absence of host-derived sequences in the phage genome. Genome annotation by Pharokka predicted 65 ORFs, all of which are protein-coding. Among these, genes encoding proteins involved in bacterial cell lysis were identified, including endolysin and holin. A whole-genome BLASTn search indicated that phage vB_PaBD_211 is in the Phikmvirus phage group, which includes *Pseudomonas* phage Ps23.FT (GenBank accession number PV641621.1) (97.96% identity) and *Pseudomonas* phage vB_Pae_PLY (GenBank accession number OR689712.1) (97.54% identity). Bacteriophage vB_PaBD_211 contains no virulence or antibiotic-resistant genes. This genome adds to the growing repository of lytic *Pseudomonas* phages relevant to the development of phage-based therapeutics.

**TABLE 1:** General features of the assembled whole genome of *Pseudomonas aeruginosa* lytic bacteriophage vB_PaBD_211

Features	*Pseudomonas aeruginosa* lytic bacteriophage vB_PaBD_211
Phage length (bp)	43,381
Coverage	570.23
GC content (%)	62.00
Completeness (CheckV) (%)	100
Contamination (CheckV) (%)	0.00
Viral genes (CheckV)	53
Quality (CheckV)	High
Host gene (CheckV)	Absent
Sequence quality	High
Life cycle	Lytic
Virulence gene	No
Antibiotic resistance gene	No

## Data Availability

The raw reads can be found at NCBI Sequence Read Archive (accession number SRX29566952). The assembly of raw reads is available at NCBI BioProject (accession number PRJNA1287049) and BioSample (accession number SAMN49806215). The complete genome sequence is available in GenBank (accession number PV786137.1).
